# Effect of the Fluorination of Graphene Nanoflake on the Dispersion and Mechanical Properties of Polypropylene Nanocomposites

**DOI:** 10.3390/nano10061171

**Published:** 2020-06-16

**Authors:** Min Gyu Lee, Sangwoon Lee, Jaehyun Cho, Seokyoung Bae, Jae Young Jho

**Affiliations:** 1 School of Chemical and Biological Engineering, Seoul National University, Seoul 08826, Korea; leemk0324@snu.ac.kr (M.G.L.); tttyyy0403@snu.ac.kr (S.L.); quartz0805@snu.ac.kr (S.B.); 2 Institute of Advanced Composite Materials, Korea Institute of Science and Technology, Jeonbuk 54896, Korea; jaehyun0119@kist.re.kr

**Keywords:** polypropylene, fluorinated graphene oxide, surface energy, dispersion, mechanical property

## Abstract

In order to investigate the effect of fluorination of graphene nanoflake on the dispersibility in polypropylene (PP) composites, fluorinated graphene oxide (FGO) was prepared by solvo-thermal reaction and applied as a filler of the PP nanocomposite. Due to the weakened inter-particle attraction among the graphene nanoflake and reduced surface energy difference between PP and the filler, PP/FGO composites showed better exfoliation and dispersion state of the filler compared with that of PP/graphene oxide (GO) or PP/reduced graphene oxide (RGO) composites. The improved exfoliation and dispersion of graphene nanoflake resulted in a significant reinforcement on the composites. The Young’s modulus and tensile strength of PP composites filled with 2 wt% of FGO increased by 31% and 15%, respectively, compared with those of PP.

## 1. Introduction

Polypropylene (PP) is one of the most abundant polyolefin and has a wide range of applications due to its low cost, good processability and recyclability. To satisfy the requirements for engineering applications, there is the need to enhance the mechanical properties of PP [1]. Polymer nanocomposites offer significant potential in the development of advanced materials with high performances and multi-functionalities. Compared with traditional composites, nanocomposites exhibit excellent mechanical, electrical and thermal properties by benefitting from the large interfacial area relative to the volume of the nanofillers [2]. Carbon materials like graphene, carbon nanotubes and fullerenes have been applied to improve the performance of polymer composites. As one of these carbon allotropes, graphene has attracted a great amount of interest due to its superior Young’s modulus, tensile strength, high electronic transport properties and thermal conductivity [3,4,5]. These properties of graphene make it a good nanofiller for polymer nanocomposites.

The dispersion of nanofillers is decisive in the mechanical properties of the polymer nanocomposites because the aggregated fillers will act as a large particle rather than nanoscale fillers [6]. Due to the hydrophobic nature and chemical inertness of the polymer chain, it is more difficult to homogeneously disperse graphene nanoflake in PP compared with in polar matrices [7]. To achieve better compatibilization between PP and the nanofiller, several attempts were focused on improving the interfacial interaction between PP matrix and graphene nanoflake such as the surface functionalization of graphene nanoflake or the chemical modification of the polymer chain [1]. For instance, surface modified graphene nanoflake with alkyl chains or PP chains improved interfacial adhesion with PP matrix [8,9,10]. PP/graphene nanoflake composites with good filler dispersion and enhanced mechanical properties were fabricated using tryptophan functionalized PP as a compatibilizer. The compatibilizer was adsorbed onto graphene nanoflake surfaces through π-π interactions between the aromatic ring of tryptophan and graphene nanoflake [11].

Another approach to improve the dispersion of graphene nanoflake in PP matrix is lowering the surface energy of the nanofiller. The van der Waals interactions among nanofillers are so strong that they tend to agglomerate in polymer matrix owing to the large surface area per unit mass. Modifying the surface of a material with fluorine can reduce its surface energy and weaken its interaction with neighboring substances. If the inter-particle attraction of nanofillers is weakened, it is easier to separate the nanofillers in the mixing process [12]. An example was reported for the PP nanocomposites with fluoro-organic modified montmorillonite. The results revealed that weakened interactions among fluoro-organic modified montmorillonite layers and lowered surface energy differences between PP and the filler promoted further exfoliation and dispersion of the filler during the melt blending [13]. Introducing C-F bonds on the graphene nanoflake surface can decrease the surface energy and make graphene nanoflake easily exfoliated with relatively weak van der Waals interaction compared with pristine graphene nanoflake [14,15]. Additionally, lowering the surface energy of graphene nanoflake reduces the polarity gap with PP. Fluorinated graphene nanoflake was shown to be well-dispersed in polymers with low surface energy such as polytetrafluoroethylene (PTFE) and polyvinylidene fluoride (PVDF) [16,17]. Therefore, the fluorination of graphene nanoflake can improve the dispersibility of the filler and its reinforcing effect on the PP nanocomposites.

In this study, we prepared PP nanocomposites filled with fluorinated graphene oxide (FGO) by melt blending. FGO was synthesized by the solvo-thermal fluorination of graphene oxide (GO) with hydrofluoric acid (HF) as a fluorine agent [18]. For comparison, reduced graphene oxide (RGO) was synthesized by the solvo-thermal reduction and blended with PP matrix [19]. This work is devoted to investigating the effect of fluorinated graphene nanoflake on its dispersibility in PP and reinforcement of the nanocomposites through the observation of the morphology and mechanical properties.

## 2. Materials and Methods

### 2.1. Materials

PP was commercial product of Lotte Chemical with the trade name of SJ-160. GO was purchased from LS-Chem (Ochang, Korea) with the trade name of GE-3550. Hydrofluoric acid (HF, 48 wt% in H_2_O) was obtained from Sigma-Aldrich (Saint Louis, MO, USA). N-methyl-2-pyrrolidone (NMP, 99.5%) was purchased from Daejung Chemicals (Siheung, Korea).

### 2.2. Preparation of PP/FGO Composites

FGO was modified by the solvo-thermal reaction between GO and HF. GO (1.0 g) was added to 300 mL of NMP and sonicated at 40 °C for 4 h. HF (20 mL) was added to the GO dispersion and the mixture was maintained at 180 °C for 6 h, 12 h and 24 h in the Teflon-lined autoclave. The autoclave was cooled to room temperature. FGO dispersion was centrifuged at 10,000 rpm for 1 h and washed with ethanol. The final product was obtained after drying in a vacuum oven at 40 °C for 24 h. The graphene nanoflakes were designated FGO_6, FGO_12 and FGO_24, according to their reaction time. For comparison, RGO was also prepared by the solvo-thermal reduction. GO (1.0 g) was added to 300 mL of NMP and sonicated at 40 °C for 4 h. GO dispersion was maintained at 200 °C for 24 h and centrifuged at 10,000 rpm for 1h. RGO was obtained after washing with ethanol and drying in a vacuum oven at 40 °C for 24 h.

The PP/graphene nanoflake composites were fabricated by melt blending using an internal mixer (MKE, Rheocomp mixer 600) with the rotor speed of 100 rpm at 200 °C for 10 min. After the melt blending, the composites were pelletized and dried at 40 °C. The amount of GO, RGO or FGOs added was 0, 0.5, 1.0 and 2.0 wt%.

### 2.3. Characterization

The thickness of RGO and FGO_12 was identified by using an atomic force microscopy (AFM, MFP-3D, Asylum Research, Santa Barbara, CA, USA) with tapping mode. The chemical composition was measured with an X-ray Photoelectron Spectroscopy (XPS, K-Alpha Spectrometer, Thermo scientific, MA, USA) using monochromatic Al Kα radiation as the exciting source. Chemical structure of each filler was analyzed by a Fourier transform infrared spectroscopy (FT-IR, Nicolet 6700, Thermo Scientific, MA, USA) in attenuated total reflection mode. The wettability of surfaces of the graphene nanoflake films was measured using the water contact angle measurement system (Smartdrop lab, Femtofab). Graphene nanoflake films were prepared by a filtering method [20]. Graphene dispersion (0.1mg/mL) was filtered through a PTFE membrane with an average pore size of 0.3 μm and washed with ethanol. The graphene film attached to the PTFE membrane was turned upside down on the glass substrate and covered with several layers of porous paper. To keep the film flat, the compressive load was applied to the membrane and the film was dried at 60 °C for 3 h in vacuum oven. After drying, the graphene nanoflake film was detached from the PTFE membrane.

The fracture surface microstructure of the composites was examined with a field emission scanning electron microscope (FE-SEM, JEOL, JSM-6701F, Tokyo, Japan). The specimens were fractured under cryogenic conditions using liquid nitrogen. The dispersion state of the GNP in the composites was observed with a three dimensional non-destructive X-ray micro-computed tomography (3D micro-CT, Skyscan 1172, Bruker, Billerica, MA, USA). The composite specimens were prepared by cutting to ca. 1.0 × 1.0 × 0.5 mm^3^. Scan data were measured with X-ray tube settings of 23 kV and 116 μA and the pixel size of 1.36 μm. The micro-CT image of the composites was obtained by reconstructing the projections.

The test specimens for measuring tensile properties were prepared using a micro injector machine (Bautek, BA-915A, Uijeongbu, Korea). The cylinder and mold temperatures were set at 200 °C and 25 °C, respectively. The tensile properties of the composites were determined using a universal testing machine (UTM, LR10K, Lloyd, West Sussex, UK) at 25 °C and at the crosshead speed of 10 mm/min according to ASTM D-638 Type V method, which is for the specimen of 63.0 × 3.2 × 3.1 mm^3^ in dimension. The thermal stability of the composites was examined by thermo-gravimetric analysis (TGA, SDT-Q600, TA Instrument, NEW, USA) at temperatures ranging from 100 to 600 °C at a heating rate of 10 °C/min under a nitrogen atmosphere.

## 3. Results and Discussion

### 3.1. Fluorination of GO

**Figure 1:**
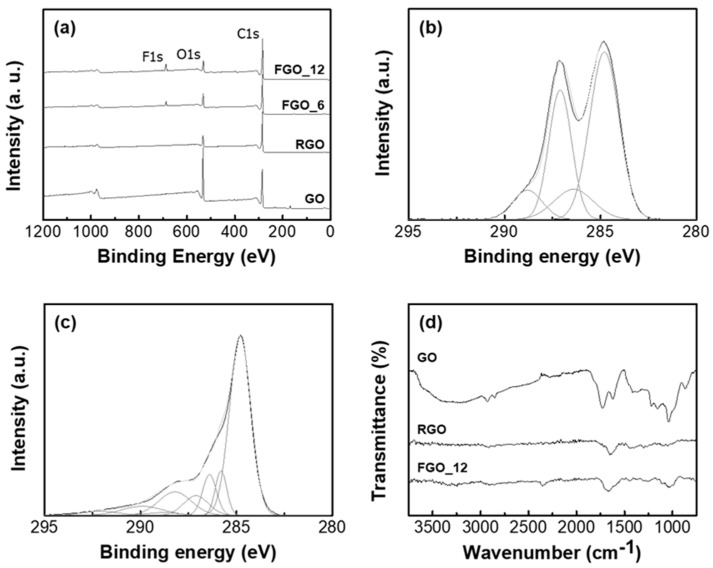
(**a**) X-ray Photoelectron Spectroscopy (XPS) spectra of graphene oxide (GO), reduced graphene oxide (RGO), fluorinated graphene oxide (FGO) _6 and FGO_12. Deconvoluted C1s XPS spectra of (**b**) GO and (**c**) FGO_12. (**d**) Fourier transform infrared (FT-IR) spectra of GO, RGO and FGO_12.

GO, RGO, FGO_6, FGO_12 and FGO_24 were analyzed by XPS to determine the elemental composition and characteristics of the chemical bonds. The quantified atomic percentages of carbon, oxygen and fluorine are presented in Table 1. As shown in Figure 1a, the oxygen content of RGO (11.7%) was clearly reduced compared with that of GO (29.1%). This showed that the solvo-thermal reduction of GO, utilizing the high boiling point of NMP, occurred. The oxygen atomic content of FGO also decreased with reaction time and a peak corresponding to fluorine was produced. C-F (covalent bond) at 687.5 eV and C-F (semi-ionic bond) at 685.5 eV in F1s spectrum was shown in Figure S1 [21,22,23]. The results indicated that oxygen-containing groups of GO were substituted with fluorine atoms by reaction between GO and HF [18]. The oxygen and fluorine atomic percentages of FGO_12 were 11.2% and 7.4%, respectively and fluorine atom per about two rings was formed on the graphene surface. FGO_24 showed the elemental composition similar to FGO_12. This was because the decrease in oxygen-containing groups lowered the reactivity. In order to observe the change in chemical composition by reduction or fluorination, deconvolution analyses of the C1s peaks in GO and FGO_12 were performed and are shown in Figure 1b,c. The C1s spectrum of GO was fitted into four peaks, C=C(sp^2^)/C-C(sp^3^) at 284.8 eV, C-OH (hydroxyl) at 286.4 eV, C-O-C (epoxy) at 287.1 eV and C=O (carbonyl and carboxylic) at 288.8 eV [24]. FGO showed a decrease in peaks associated with oxygen-containing groups but had additional peaks contributed by C-C-F, C-F, CF-CF_2_ and CF_2_ at 285.8, 288.2, 289.9 and 292.1, respectively [25,26]. These results indicated that the oxygen-containing groups were effectively substituted by fluorine elements.

Fourier transform infrared spectroscopy (FT-IR) was used to further investigate the effect of reduction and fluorination and the results are shown in Figure 1d. In the spectrum of GO, peaks from the stretching and bending of O-H bonds by hydroxyl groups were observed at 3000–3700 cm^−1^ and 1625 cm^−1^, respectively [27]. A peak attributed to the stretching of C=O bonds of carboxyl groups was shown at 1730 cm^−1^ and stretching peaks from C-O bonds of carboxyl, epoxy and hydroxyl groups were observed at 1380, 1200 and 1040 cm^−1^, respectively [28]. In the case of RGO, the peaks corresponding to the oxygen-containing group apparently decreased due to the reduction. Compared with RGO, FGO_12 exhibited a broad band attributing to the stretching of C-F bonds at 1000–1250 cm^−1^. The same result was observed in other fluorinated graphene nanoflake studies because the C-F bonds vary from covalent bonds, through semi-ionic bonds, to ionic bonds. This is due to the large electronegativity difference between carbon and fluorine atoms [25,29].

**Figure 2:**
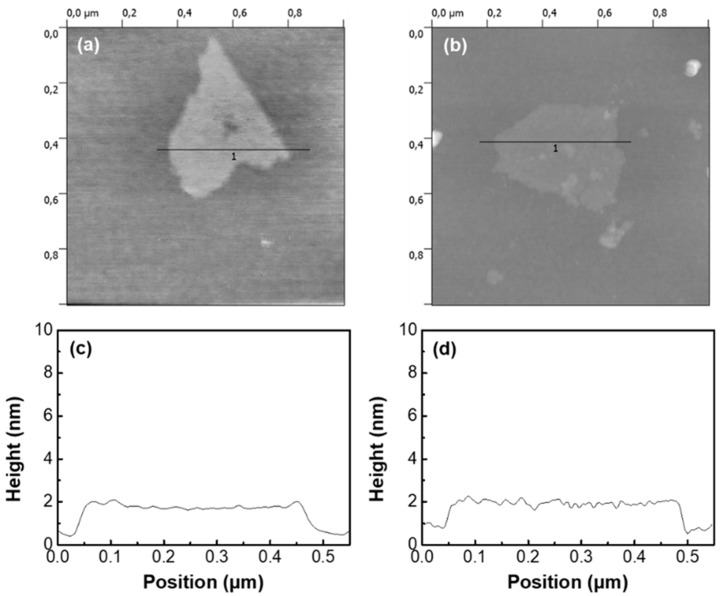
Atomic force microscopy (AFM) images of (**a**) RGO and (**b**) FGO_12 on a Si substrate. The height profiles of (**c**) RGO and (**d**) FGO_12.

The thickness of RGO and FGO_12 was identified by AFM tapping mode (Figure 2). Both the thickness of RGO and FGO was observed to be around 2 nm, which means that re-aggregation of the graphene nanoflake did not occur after the reduction or fluorination and the exfoliation was well maintained during the process.

**Figure 3:**
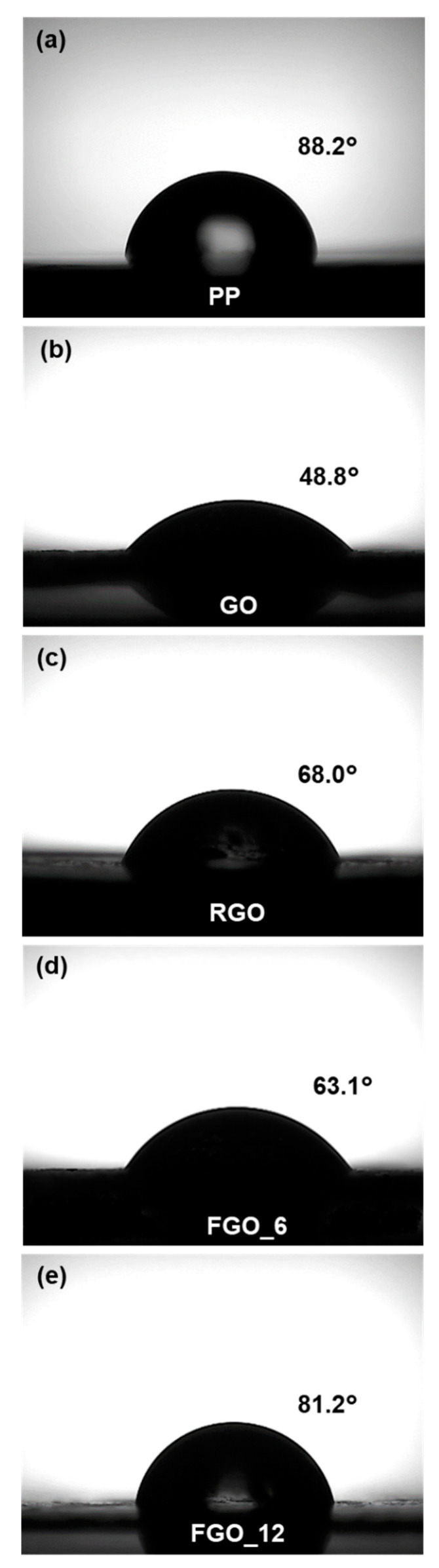
Optical images of probe liquid droplets on the (**a**) polypropylene (PP), (**b**) GO, (**c**) RGO, (**d**) FGO_6 and (**e**) FGO_12 film surfaces.

Optical images of the probe droplets of PP, GO, RGO, FGO_6 and FGO_12 observed with the water contact angle measurement were shown in Figure 3. GO, which has good affinity with water due to its hydrophilic groups on the surface, exhibited a low contact angle of 48.8°. As the hydrophobicity increased due to the reduction of the oxygen functional groups, the contact angle of the RGO increased to 68.0°. In case of FGOs, the contact angle was increased due to the substitution of the oxygen-containing groups with fluorine atoms and FGO_12 showed a contact angle of 81.2°. The surface energies of GO, RGO, FGO_6 and FGO_12 were determined to be 53.4, 42.0, 50.0 and 33.8 mJ/m^2^, respectively. The similar result that C-F bonds reduced the surface energy of graphene nanoflake was reported [30]. FGO_12 had the closest surface energy value to 29.1 mJ/m^2^, the surface energy of PP.

### 3.2. Morphology of PP/Graphene Nanoflake Composites

**Figure 4:**
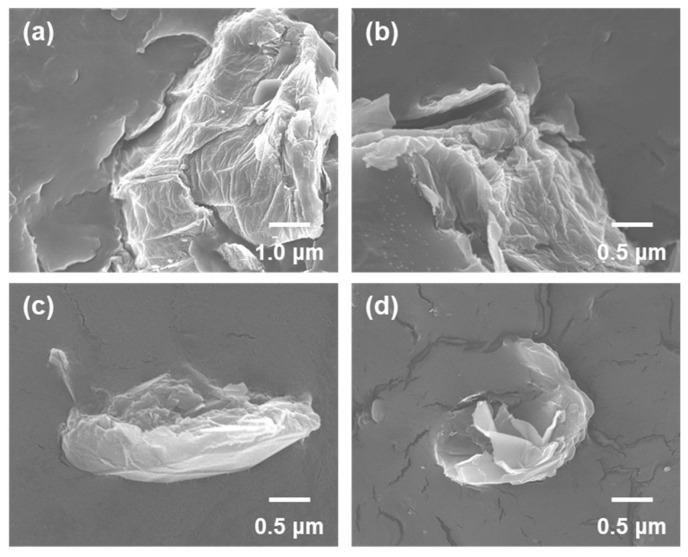
Scanning electron microscopy (SEM) micrographs of the cryofracture of (**a**) PP/GO, (**b**) PP/RGO, (**c**) PP/FGO_6 and (**d**) PP/FGO_12 composites.

**Figure 5:**
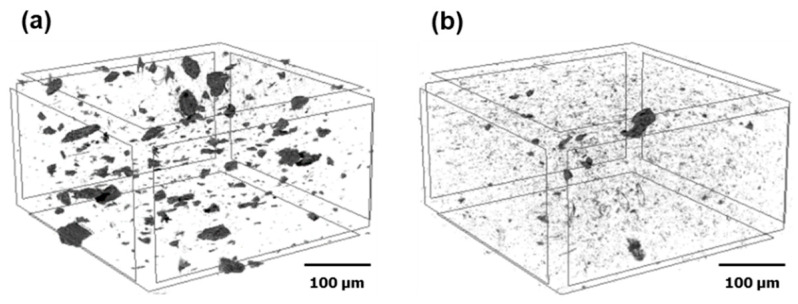
Three dimensional non-destructive X-ray micro-computed tomography (3D micro-CT) images of (**a**) PP/RGO and (**b**) PP/FGO_12 composites.

The fracture surfaces of PP/GO, PP/RGO, PP/FGO_6 and PP/FGO_12 composites were examined. The content of graphene nanoflakes in the composite specimens was 2 wt%. SEM images of Figure 4 showed the difference in the size and exfoliation state of the graphene nanoflake in the composites. GO was observed in large aggregates as a result of its poor compatibility to PP due to the large surface energy difference. RGO existed in the similar stacked form as GO despite the smaller surface energy difference from PP. This meant the surface energy was still too high, facilitating the tendency of nanofiller to aggregate strongly. On the other hand, FGO_12 existed inside the composite in the form of thin sheets. As the surface energy of FGO_12 was reduced to the similar level of PP, the cohesion among fillers was weakened and the dispersion and exfoliation of the filler were improved during the melt blending. As shown in Figure 5, The overall dispersion state of graphene nanoflakes in the composites observed by 3D-micro CT also showed better dispersion of FGO_12 compared with RGO. 3D micro-CT analysis has been used for observing the internal structure of polymer nanocomposites without destroying the specimens [31,32]. It could be concluded that the weakened inter-particle interaction of the nanofiller and reduced surface energy difference with PP matrix promoted the dispersibility of the graphene nanoflake [14].

### 3.3. Mechanical and Thermal Properties of PP/Graphene Nanoflake Composites

**Figure 6:**
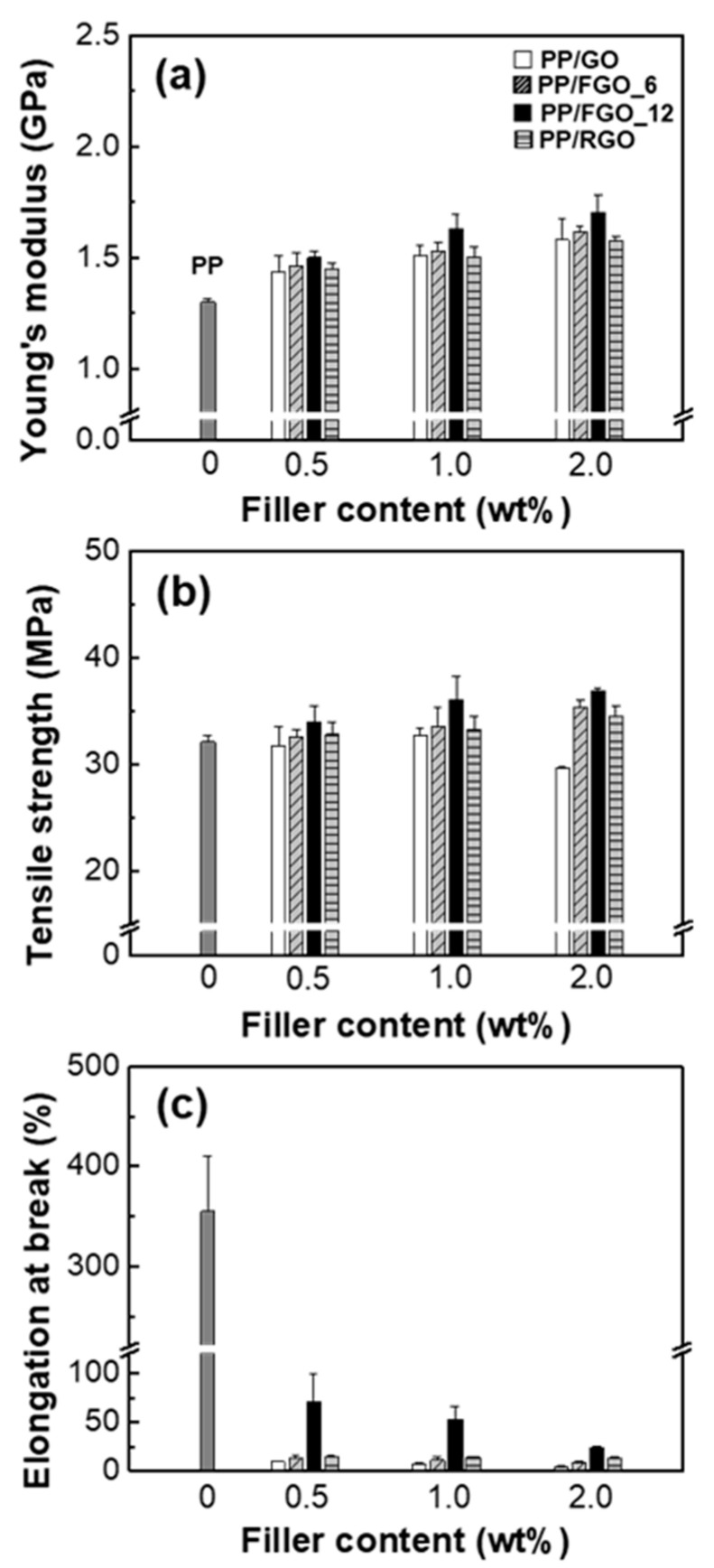
Mechanical properties measurement: (**a**) Young’s modulus, (**b**) tensile strength and (**c**) elongation at break of PP, PP/GO, PP/FGO_6, PP/FGO_12 and PP/RGO composites as a function of filler content.

Figure 6 showed the tensile properties of PP composites with varying amount of graphene nanoflake. The Young’s modulus of PP/FGO composites was more effectively improved compared with that of PP/GO composites. FGOs which showed better dispersion and exfoliation from morphological observations resulted in more effective stress transfer by higher aspect ratios and wider interfaces with the polymer matrix [33,34,35]. PP/RGO composites showed similar Young’s modulus to PP/GO composites because the filler still existed in aggregated forms. PP/GO composites showed little increase in tensile strength due to the poor dispersion and interfacial adhesion from the high surface energy differences between the matrix and the filler. The tensile strength decreased with 2 wt% of GO as the fracture occurred before the yield point. The highest Young’s modulus and tensile strength were shown in PP/FGO_12 composite at 2 wt% of filler loading, which increased 31% and 15% compared with those of PP, respectively. While the decreased surface energy of graphene nanoflake improved their dispersibility in PP matrix, the interfacial adhesion between the matrix and the filler was not affected as good as filler dispersibility. This is because there was no expected interaction between FGO and PP. Thus the stress transfer between the matrix and the filler was not effective considering the filler dispersion. However, unlike the tensile strength, Young’s modulus is little affected by this as it is measured in very low deformation stage and is mainly affected by filler content and dispersion state [31,36]. Therefore, it can be explained that the improvement is more noticeable in the Young’s modulus than tensile strength in PP/FGO composites [13]. The elongation at break of the composites drastically decreased compared with that of PP because the rigid nanofillers restricted the polymer chain mobility and defects existing at the interface acted as stress concentrators. Nevertheless, PP/FGO_12 composites showed relatively high elongation compared with other composites due to the good dispersibility of the filler [37]. The fluorination of graphene nanoflake homogeneously dispersed the nanofiller in PP matrix similar to other approaches focusing on the improvement of interfacial interaction between the matrix and the filler. However, fluorinated graphene nanoflake had a disadvantage in terms of interfacial adhesion, limiting the enhancement of the tensile strength. A method should be devised that can consider the interaction with PP while lowering the surface energy of the nanofiller. The method can be expected to further improve the mechanical properties of the PP nanocomposites.

The thermal stability of PP and the composites filled with 2 wt% of graphene nanoflakes was examined by determining the weight loss during heating. Figure S2 showed the weight loss curves of the specimens at the heating rate of 10 °C/min. The temperatures for 5 % weight loss (T_5%_) of PP, PP/GO, PP/RGO and PP/FGO_12 were 347, 383, 382 and 385 °C, respectively. The PP composites exhibited improved thermal stability at the initial stage of degradation compared with PP because the fillers act as heat sink that prevents heat from accumulating in PP. The temperatures at which the thermal degradation ends and there is no temperature change (T_end_) of PP, PP/GO, PP/RGO and PP/FGO_12 were 427, 463, 468 and 484 °C, respectively. The difference in T_end_ between PP/FGO and PP/RGO was larger than the difference of T_5%_ between PP/FGO and PP/RGO, which means the enhancement of the resistance to thermal degradation. This is because graphene nanoflakes also serve as transfer barrier and FGO with the improved dispersion more effectively hindered the volatile decomposed products [4].

## 4. Conclusions

Due to the high surface energy difference between PP and fillers, PP/GO and PP/RGO composites showed the poor dispersibility of graphene nanoflake. Thus, the composites showed little improvement in mechanical properties despite the increase of the filler content and a sharp drop in elongation at break. In order to reduce the surface energy of graphene nanoflake, fluorinated graphene nanoflake was prepared by solvo-thermal reaction between GO and HF. Through the observation of the morphology by SEM and 3D-micro CT, the improved exfoliation and dispersion of the filler were confirmed in the PP matrix. Since exfoliated graphene nanoflake had high aspect ratios, achieving effective stress transfer in polymer composites, PP/FGO composites exhibited better mechanical properties compared with those of PP/GO or PP/RGO composites. It was certain that the weakened inter-particle interaction of graphene nanoflake and reduced surface energy difference with PP achieved the improved dispersibility of the nanofiller and enhanced mechanical properties of the composites.

## Figures and Tables

**Table 1:** Atomic percentages of GO, RGO, FGO_6, FGO_12 and FGO_24.

Sample	Carbon (%)	Oxygen (%)	Fluorine (%)
GO	70.9	29.1	-
RGO	88.3	11.7	-
FGO_6	80.0	16.7	3.3
FGO_12	81.4	11.2	7.4
FGO_24	82.5	10.4	7.1

## Supplementary Materials

The following are available online at https://www.mdpi.com/2079-4991/10/6/1171/s1, Figure S1: F1s XPS spectra of FGO_12, Figure S2: TGA data of PP, PP/GO, PP/RGO and PP/FGO_12 composites.
