# Supplementary material for: Effect of the Fluorination of Graphene Nanoflake on the Dispersion and Mechanical Properties of Polypropylene Nanocomposites
Source: Nanomaterials (Basel). 2020 Jun 16;10(6):1171. doi: 10.3390/nano10061171 (PMC7353096; doi:10.3390/nano10061171)
Supplement: Supplementary file 1 [file nanomaterials-10-01171-s001.pdf]

# Supplementary Materials

# Effect of the fluorination of graphene nanoflake on the dispersion and mechanical properties of polypropylene nanocomposites

**Min Gyu Lee [1], Sangwoon Lee [1], Jaehyun Cho [2], Seokyoung Bae [1], and Jae Young Jho [1,*]**

[1] School of Chemical and Biological Engineering, Seoul National University, Seoul 08826, South Korea; leemk0324@snu.ac.kr

[2] Institute of Advanced Composite Materials, Korea Institute of Science and Technology, Jeonbuk 54896, South Korea; jaehyun0119@kist.re.kr

[*] Correspondence: jyjho@snu.ac.kr; Tel.: 82-2-880-8346

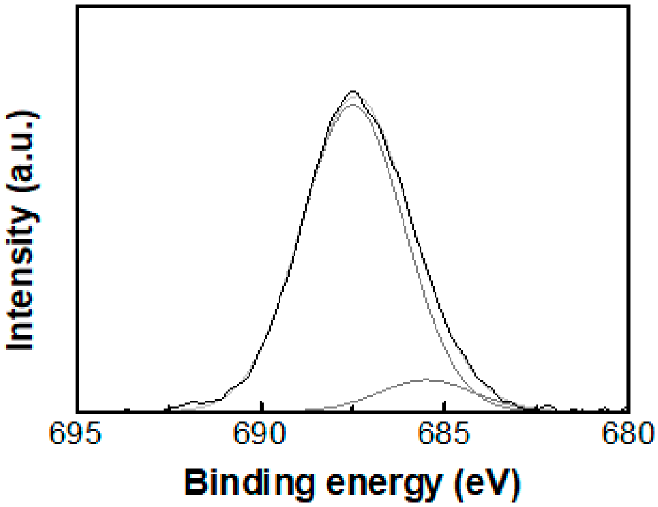


**Figure S1.** F1s XPS spectra of FGO_12.

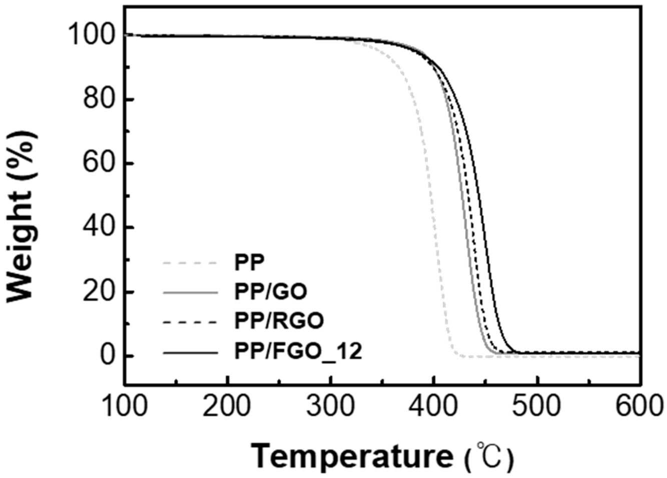


**Figure S2.** TGA data of PP, PP/GO, PP/RGO, and PP/FGO_12 composites.
